# Vaccination

**Published:** 1882-03

**Authors:** 


### VACCINATION.

The utility of vaccination, and its safety, is doubted, at this time, only by the professional crank or the illy-informed layman. It is the duty of the profession to endeavor to hasten the day when an unprotected person can be found only in uncivilized communities—and as rarely as possible there.

At the risk of dealing with a subject that may have grown stale to some, we must urge some thoughts which we deem important in connection therewith :

First, we submit that thorough vaccination means *saturation* of the system with the vaccine disease. Variola may be reduced to varioloid by *partial* saturation. Varioloid could, in most cases, be reduced to nothing by *complete* saturation. This would be a vast gain ; for while varioloid may be comparatively harmless to the individual, it may be terribly serious to the community.

We claim that science knows no method by which this saturation can be ascertained but by revaccination. Neither a large "scar" nor a small one, neither a smooth one nor a pitted one, old or recent, nor one nor a dozen, can afford the information gained only by revaccination. We occasionally find that revaccination fails when the scar is single and scarcely perceptible, and so old as to have almost lost its history. Again, it proves successful when several not very old marks may thrust themselves upon our view. The present fashionable idea that several marks are essential is not wholly true; and that they are positive proof of complete protection is none the less a mistake. If these different marks have been made at different periods, then the value of their testimony is enhanced. In fine, revaccination is the test, the only test--such revaccination as brings fresh virus in contact with a surface that will absorb it. There is an idea that vaccination "runs out" very abruptly and unceremoniously at a certain time; the mystic seven years being the time commonly selected for such short-stop. Rather than seven years, let us say seven months, or weeks, or days, if saturation was not attained. If such a saturation was achieved, then it may be a life-time; but to be satisfied of such completeness, and to be on the safe side, an occasional dose of the virus, where time and occasion suffice, will serve to make all parties more comfortable.

What virus shall be used? Bovine is now the rage. Nor do we object, except that it is more expensive, sometimes not procurable, and occasionally disappoints. This comes either from age of the article or from lack of care in dissolving and applying it. It usually differs but little in effect from the humanized; though we have observed instances in which the attending inflammation was severe, and marked by the characteristic eruption over a large part of the arm. We are ready to say that it is well to make revaccination (where the humanized was used in the first instance) with the bovine.

We are not ready, though, to desert our old-time friend, good humanized virus. True, we want to know from whom it is derived, and if a crust, how it was preserved, whether the vesicle was injured or suppuration occurred. We want a crust of inspissated lymph, not a scab of dried pus. Dried pus is not vaccine lymph or virus; it is not only inefficient for protection, but may be actually deleterious in its consequences.

It should be remembered, too, that a vaccine crust is a short-lived animal—if there be about it enough of the bacteria or other microscopical beast to call it an animal. A very few weeks ends its vitality, even though it be kept from heat, light and air. If carried in the pocket, the heat from the body will probably precipitate its destruction. At one time we fondly indulged the hope that the lymph, hermetically sealed in capillary glass tubes, had solved the problem; but we were sadly mistaken. Quill points may be dipped in the fresh lymph and are convenient; but only for a few days.

Is there serious danger of transmitting other diseases with vaccinia? We believe not. The contrary view is popular, and has given bovine virus its present prominance. Syphilis is the great bug-bear with which non-believers fight vaccination. Non-specific skin diseases come in as of secondary importance. Scrofula and tubercle also erect their grim and grisly form before such affrighted innocents. We have never known of a case of syphilis communicated by vaccination. It is probably impossible to communicate any of these affections, except the *blood* be included in the lymph or crust, and we are not so very sure it is often possible in such case. It is pretty well ascertained that pathological secretions, not containing pus or blood, do not convey constitutional disease. Vaccine lymph is a pathological secretion (if secretion it can be called).

Then, let us conclude:

(1.) Bovine virus, fresh and pure, is good and desirable.

(2.) Humanized virus, fresh and pure, is good and desirable.

(3.) Vaccinate early and often.

